# Tyrosine kinase receptor TIE-1 mediates platinum resistance by promoting nucleotide excision repair in ovarian cancer

**DOI:** 10.1038/s41598-018-31069-2

**Published:** 2018-09-04

**Authors:** Masumi Ishibashi, Masafumi Toyoshima, Xuewei Zhang, Junko Hasegawa-Minato, Shogo Shigeta, Toshinori Usui, Christopher J. Kemp, Carla Grandori, Kazuyuki Kitatani, Nobuo Yaegashi

**Affiliations:** 1 Department of Obstetrics and Gynecology, Tohoku University Graduate School of Medicine, Sendai, Japan; 2 Tohoku Medical Megabank Organization, Tohoku University Graduate School of Medicine, Sendai, Japan; 3 Division of Human Biology, Fred Hutchinson Cancer Research Center, Seattle, WA USA; 4 SEngine Precision Medicine, Seattle, WA USA; 5 Laboratory of Immunopharmacology, Faculty of Pharmaceutical Sciences, Setsunan University, Neyagawa, Japan

## Abstract

Platinum resistance is one of the most challenging problems in ovarian cancer treatment. High-throughput functional siRNA screening identified tyrosine kinase with immunoglobulin-like and EGF-like domains 1 (TIE-1) as a gene that confers cells resistant to cisplatin. Conversely enforced over-expression of TIE-1 was validated to decrease cisplatin sensitivity in multiple ovarian cancer cell lines and up-regulation of TIE-1 was correlated with poor prognosis and cisplatin resistance in patients with ovarian cancer. Mechanistically, TIE-1 up-regulates the nucleotide excision repair (NER) system mediated by xeroderma pigmentosum complementation group C (XPC), thereby leading to decreased susceptibility to cisplatin-induced cell death without affecting cisplatin uptake and excretion. Importantly potentiation of therapeutic efficacy by TIE-1 inhibition was selective to DNA-adduct-type chemotherapeutic platinum reagents. Therefore, TIE-1 is suggested to promote XPC-dependent NER, rendering ovarian cancer cells resistant to platinum. Accompanied with novel findings, TIE-1 could represent as a novel therapeutic target for platinum-resistant ovarian cancer.

## Introduction

Ovarian cancer is the eighth most common cancer among women and the most lethal gynecological cancer, and an estimated 239,000 new cases and 152,000 deaths were reported worldwide in 2012^1^. Platinum complexes play a central role as the first-line treatment option for ovarian cancer, and are usually administered in combination with taxanes. Although ovarian cancer is a relatively chemo-sensitive disease, around 20–30% of patients are refractory to platinum-based chemotherapy^2,3^. Moreover, even after effective clearance of tumor cells in response to standard therapy, many patients (70–90%) relapse within months to years, and the tumors relapsed within 6 months are typically resistant to platinum^4^. Identification of the molecular mechanisms responsible for platinum resistance is thus urgently required to improve treatments for patients with refractory ovarian cancer.

Cisplatin is a platinum coordination compound that becomes active once it enters the cell, actively and/or passively. Activated cisplatin almost exclusively forms intra-strand platinum-DNA crosslinks and causes DNA damage^5^, blocking cell division and resulting in apoptotic cell death. A wide range of chemo-resistant mechanisms have been identified, including down-regulation of cisplatin uptake^6^, up-regulation of cisplatin excretion^7^ and detoxification^8^, down-regulation of ceramide-mediated apoptosis^9^, and increased repair or tolerance of DNA damage^10,11^. Although numerous genes have been implicated in chemo-resistance^12^, the key molecules mediating chemo-resistance remain to be identified.

RNA interference (RNAi) high-throughput screening has the potential to identify novel genes responsible for specific cell functions and has been employed to identify key determinants of drug sensitivity^13–16^. Salm *et al*. identified a key molecule mediating cisplatin resistance in neuroblastoma by RNAi screening of 719 known proteins in the human genome^17^. High-throughput screening targeting genome-wide molecules thus provides a highly effective research tool for investigating key determinants of cisplatin-resistance.

In the present study, we used genome-wide siRNA libraries covering over 6659 genes and identified tyrosine kinase with immunoglobulin-like and EGF-like domains 1 (TIE-1) as a novel gene mediating cisplatin resistance in ovarian cancer cells. TIE-1 has been shown to play a critical role in angiogenesis and blood-vessel stability by inhibiting angiopoietin 1 signaling through the endothelial receptor tyrosine kinase receptor TIE-2. However, TIE-1 pathobiology in ovarian cancer remains unknown. We further explored the molecular mechanisms by which TIE-1 mediates cisplatin resistance.

## Results

### Functional Genome-wide siRNA Screening Identified TIE-1 as a Gene Mediating Cisplatin Resistance in Ovarian Cancer Cells

**Figure 1:**
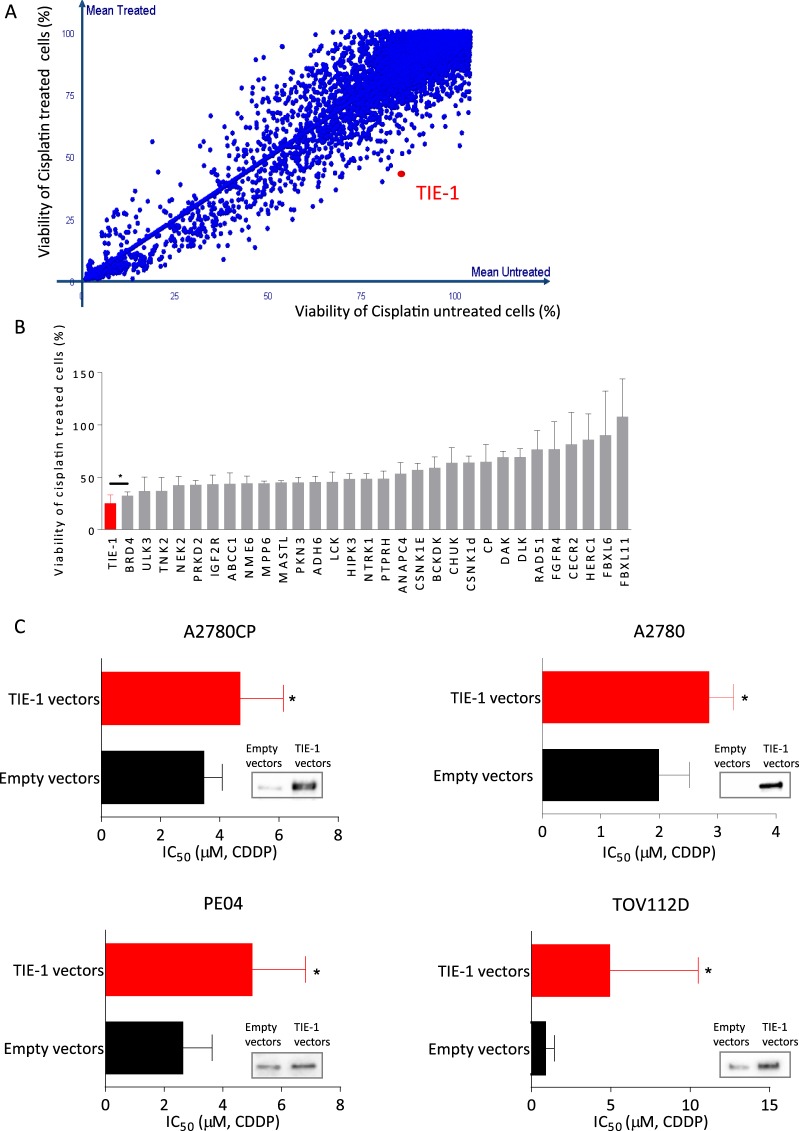
Functional high-throughput siRNA screening of therapeutic target genes in cisplatin-resistant ovarian cancer cells. (**A**) Cisplatin-resistant A2780CP cells were plated onto 384-well plates for high-throughput siRNA screening. Cell viabilities of cisplatin-treated (y-axis) and untreated (x-axis) cells relative to control siRNA treatment were shown. Viability on the Y axis is normalized relative to viability of mock transfected cisplatin treated cells. Genes which enhance toxicity of cisplatin after siRNA knockdown are located to the right of the diagonal. (**B**) Validation of 30 potential hit genes from high-throughput screen with specific siRNAs. Cells were transfected with control siRNA or gene-specific siRNAs followed by treatment with 3 µM cisplatin for 72 h. Cell viability in control-siRNA-treated cells decreased by 56% after cisplatin treatment. Values indicate the percentage of remaining cell viability in gene-specific siRNAs compared to control siRNA. Two independent experiments were performed. (**C)** A2780CP cells (2 × 10^3^ cells/well) were transfected with empty or TIE-1 vectors for 24 h and cellular proteins were extracted and submitted to immunoblotting with TIE-1 antibody. (**D**) Ovarian cancer cells (A2780, A2780CP, PE04, and TOV112D) were plated in 96-well plates, transfected with empty vector or TIE-1 vector for 24 h, and then treated with cisplatin for 72 h. Cell viability was determined by Celltiter-Glo assay. IC_50_ values represent mean ± SD of three independent experiments. *P < 0.05. For the cropped blots, protein samples were run under same conditional treatments and processed in parallel. Full-length blots are presented in Supplementary Fig. S9.

Functional high-throughput screening using small interfering RNAs (siRNAs) specific to 6659 genes was performed in cisplatin-resistant A2780CP ovarian cancer cells to identify genes, the knockdown of which potentiated cisplatin inhibition of cell growth. A2780CP cells transfected with siRNAs were treated with cisplatin and cell viability was determined. The viability of cisplatin-treated (y-axis) and untreated (x-axis) cells compared to control siRNA is shown in Fig. 1(A). Knockdown of 320 subset genes significantly increased the susceptibility of A2780CP cells to cisplatin-induced loss of cell viability (the entire list of 320 hit genes are shown in the manuscript in preparation by de Leeuw *et al*.). Herein we performed the validation of 30 of the sensitizer genes using newly synthesized siRNA sequences and specific knockdown of orphan tyrosine kinase receptor TIE-1 resulted in the greatest increase of cisplatin-mediated cytotoxicity (Fig. 1(B)).

Reciprocally, we constructed a human TIE-1 expression vector and determined the effects of TIE-1 over-expression on chemo-sensitivities of ovarian cancer cells. Its over-expression significantly increased inhibitory concentration_50_ (IC_50_) values for cisplatin in multiple ovarian cancer cell lines, including cisplatin-resistant A2780CP and PE04^18^ cells as well as cisplatin-sensitive A2780 and TOV112D cells. The IC_50_ values of empty vectors and TIE-1-transfected cells were as follows: 3.46 and 4.68 µM (A2780CP), 2.63 and 4.99 µM (PE04), 1.61 and 2.61 µM (A2780), and 0.84 and 4.94 µM (TOV112D) (Fig. 1(C)). These data suggest that TIE-1 is a candidate cisplatin resistance gene in ovarian cancer cells.

### High Expression of TIE-1 Correlates with Poor Prognosis and Cisplatin Resistance in Patients with Ovarian Cancer

**Figure 2:**
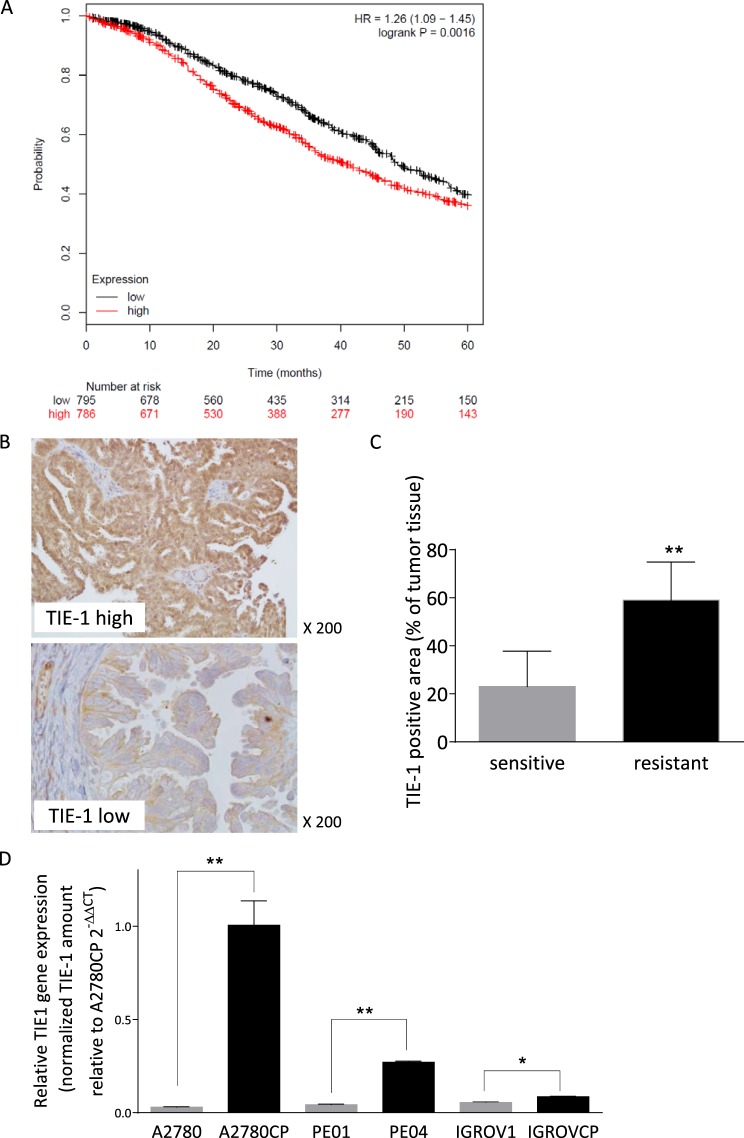
High expression of TIE-1 correlates with poor prognosis and cisplatin-resistance. (**A**) A data set of 1561 normalized ovarian cancer microarrays was downloaded from the Kaplan– Meier Plotter database and overall survival curves for high expression (defined as cancers with values above the median) or low expression were analyzed. (**B**) Representative images of TIE-1 immunoreactivity in ovarian cancer tissues before cisplatin treatment (×200 magnification). Upper or lower image represent TIE-1-high or -low expression areas of ovarian cancer tissues, respectively. (**C**) Percentage TIE-1 positive area in total cancer-tissue at pre-cisplatin treatment. Total cancer-tissue area and TIE-1-positive area were counted manually in five randomly selected high-power fields. The percentage of TIE-1-positive area was determined for each sample. Tissues from 5 platinum-sensitive and 8 platinum-resistant patients were examined. *P* values were determined by paired *t*-tests (two-sided). Values represent the mean ± SD. *P < 0.05. (**D**) TIE-1 mRNA expression levels in cisplatin-resistant and -sensitive parental cells were determined by quantitative real-time PCR. *P < 0.05, **P < 0.01.

Drug resistance is a key factor determining patient prognosis in ovarian cancer. To determine the clinical significance of TIE-1 expression in ovarian cancer patients, we investigated the correlation between TIE-1 expression and patient prognosis. Analysis of the public database (KM plotter: http://kmplot.com/) showed that high TIE-1 expression was significantly correlated with poor prognosis in ovarian cancer (n = 1561) (Fig. 2(A)). Even in the group of patients with platinum-based chemotherapy (n = 1409), TIE-1 high expression was also significantly correlated with poor prognosis (Supplementary Fig. S1), suggesting a possible involvement of TIE-1 up-regulation in the acquisition of platinum resistance in ovarian cancer cells.

The ovarian cancer tissues were obtained from the 13 patients who underwent laparotomy before platinum-treatment, and TIE-1 immunohistochemistry in the tumor tissues were performed. TIE-1 high expressing areas and low expressing areas were observed in a single patient. The area of TIE-1 high expression in individual patients (n = 13) was quantified. According to clinical characteristics, we sub-grouped patients to chemo-resistant (n = 8) and chemo-sensitive (n = 5). Total cancer-tissue area and TIE-1-positive area were counted manually in five randomly selected high-power fields. Interestingly, immunohistochemistry of TIE-1 in ovarian cancer tissues (Fig. 2(B)) revealed that TIE-1-positive tumor areas were significantly increased in platinum-resistant patient group (59%) compared to platinum-sensitive group (23%; Fig. 2(C)) (patient characteristics are shown in Supplementary Table 1).

We also examined the association between TIE-1 expression and cisplatin resistance in 3 paired ovarian cancer cell lines. TIE-1 mRNA levels were higher in cisplatin-resistant lines compared with naive parental cells (Fig. 2(D)). Protein levels of TIE-1 were correlated with the IC_50_ values of each cell lines (Supplementary Fig. S2).

These results suggest TIE-1 is involved in platinum-resistance of ovarian cancer.

### TIE-1 Promotes DNA Damage Repair

**Figure 3:**
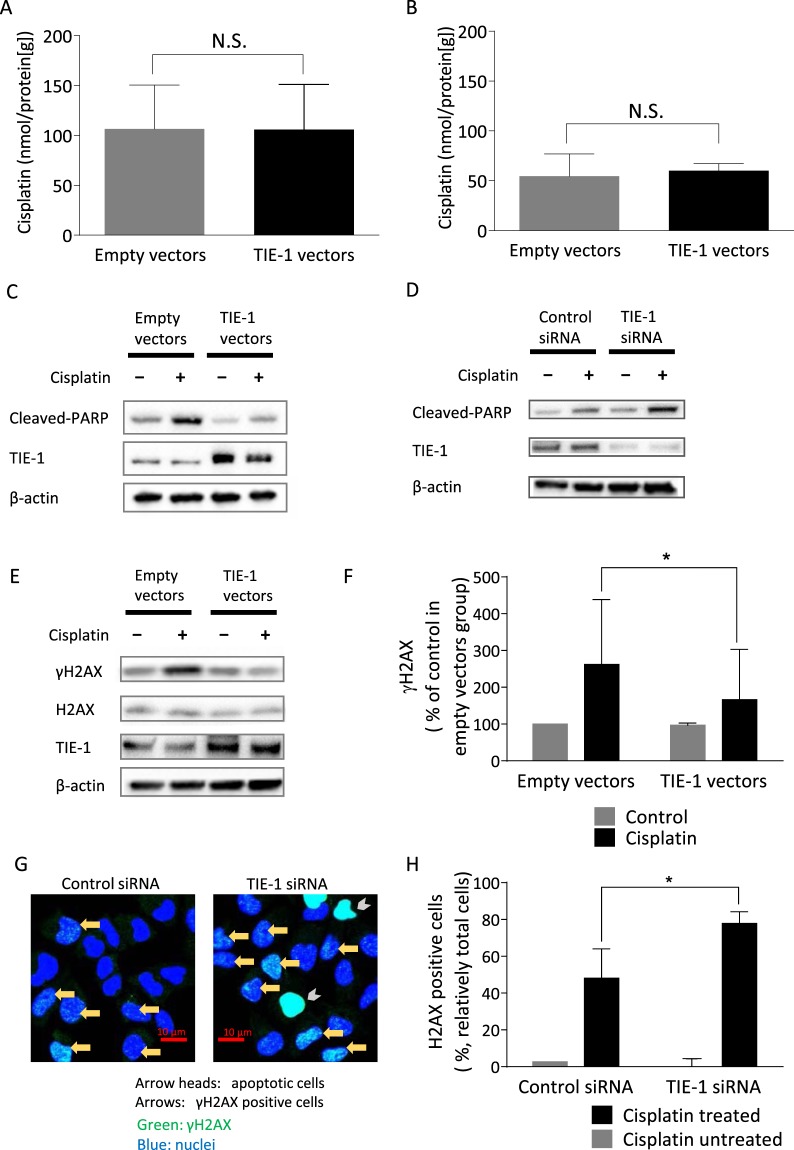
Roles of TIE-1 in cisplatin pharmacodynamics and pharmacokinetics. A2780CP cells were transfected with empty or TIE-1 vectors, and treated 24 h later with 10 µM cisplatin for 3 h. Intracellular cisplatin concentrations were measured to determine cisplatin uptake (**A**). Cisplatin excretion (**B**) was determined after replacing the culture medium with cisplatin-free medium and incubating the cells for a further 3 h. After the culture medium was washed out, the cells were harvested, and cellular cisplatin levels were determined. (**C**,**D**) A2780CP cells were transfected with empty or TIE-1 vectors (**C**), control or TIE-1 siRNAs (**D**) for 24 h and then treated with 10 µM cisplatin for 12 h. Extracted proteins were submitted to immunoblot analysis using antibodies specific for cleaved PARP, TIE-1, and β-actin. Results are representative of three independent experiments. For the cropped blots, protein samples were run under same conditional treatments and processed in parallel. Full-length blots are presented in Supplementary Figs S10, S11. (**E**,**F**) A2780CP cells were transfected with empty or TIE-1 vectors for 24 h followed by treatment with 10 µM cisplatin or PBS. Extracted proteins were submitted to immunoblot analysis using antibodies specific for γH2AX, H2AX, TIE-1, and β-actin. Results are representative of three independent experiments. For the cropped blots, protein samples were run under same conditional treatments and processed in parallel. Full-length blots are presented in Supplementary Fig. S12(E). γH2AX levels were quantified. Values represent the mean ± SD. *P < 0.05 (**F**). (**G**,**H**) γH2AX expression was evaluated by immunofluorescence microscopy. A2780CP cells plated on glass bottom 35 mm dishes were transfected with control or TIE-1 siRNAs for 24 h and then treated with 10 µM cisplatin or PBS for 12 h. After treatment, cells were stained with Hoechst 33342 and γH2AX-specific antibody. The cells having detectable pleural foci in nucleus are determined as γH2AX positive. Representative images are shown (**G**). The numbers of γH2AX positive cells were counted and the results are representative of three independent experiments (**H**). Values represent the mean ± SD. *P < 0.05.

Up-regulation of cisplatin excretion through multidrug-resistance transporters contributes to platinum-resistance^7^. To determine if TIE-1 modulated cisplatin influx/efflux, we evaluated intracellular platinum levels by atomic absorption spectrometry. TIE-1 over-expression had no effect on cisplatin influx or efflux (Fig. 3(A,B)), thus showing less involvement of TIE-1 in cisplatin pharmacokinetics at the cellular level.

Cisplatin is known as a DNA-damaging reagent that induces apoptosis in cancer cells. TIE-1 over-expression significantly inhibited poly-ADP-ribose polymerase (PARP) cleavage induced by cisplatin, while TIE-1 knock-down potentiated it (Fig. 3(C,D)). These results indicate that TIE-1 had an inhibitory effect on cisplatin-induced apoptosis. This cisplatin-induced apoptotic cell death results from DNA damage. We determined the effect of TIE-1 on DNA damage responses. A2780CP cells treated with cisplatin for 12 hours showed an obvious accumulation of γH2AX as a marker of DNA damage (Supplementary Fig. S3). TIE-1 over-expression significantly suppressed the formation of γH2AX in cisplatin-treated cells (Fig. 3(E,F)). Reciprocally, TIE-1 knockdown promoted γH2AX accumulation induced by cisplatin (Fig. 3(G,H)).

These results suggest that TIE-1 serve as a DNA damage-protective protein, possibly by promoting DNA repair.

### TIE-1 Promotes Nucleotide Excision Repair (NER) through Xeroderma Pigmentosum Complementation Group C

Cross-linkage of DNA bases formed by **c**isplatin adducts are repaired by the NER system^13^, which consists of four steps: damage recognition, verification and helix opening, excision, and gap filling^19,20^. We first focused on the damage verification step to determine if TIE-1 up-regulated the NER system.

**Figure 4:**
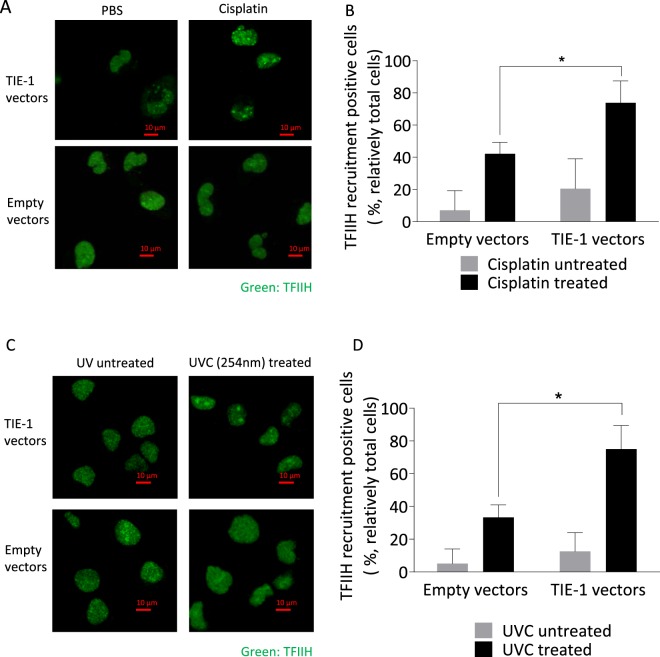
TIE-1 increases NER. (**A**) Recruitment of TFIIH to damaged DNA was examined by confocal microscopy. TOV112D cells transfected with empty or TIE-1 vectors were treated with 10 µM cisplatin for 1 h, fixed with 4% formaldehyde, and stained with TFIIH antibodies. The numbers of TFIIH recruitment positive cells were counted. Experiments were repeated three times independently and representative images are shown. (**B**) Values represent the mean ± SD. *P < 0.05. (**C**) Recruitment of TFIIH to UV irradiation-induced DNA damage. TOV112D cells transfected empty or TIE-1 vectors were exposed UVC 100 J/m. Following 5 minutes after irradiation, cells were fixed with 4% formaldehyde, and stained with TFIIH antibodies. The numbers of TFIIH recruitment positive cells were counted and the results are representative of three independent experiments. (**D**) Values represent the mean ± SD. *P < 0.05.

Transcription factor II human (TFIIH) is responsible for DNA-damage verification and helix opening and is recruited to form a complex^19,20^, thereby serving as a marker for NER. Cellular TFIIH localization was visualized by immunofluorescence microscopy. TFIIH was re-localized to the nucleus in response to cisplatin-induced DNA damage. Importantly, TIE-1 over-expression increased TFIIH re-localization by cisplatin treatment (Fig. 4(A,B)). It is well known that UV irradiation-induced DNA damage is repaired by NER. TIE-1 over-expression increased TFIIH re-localization by UV irradiation (Fig. 4(C,D)). TIE-1 was confirmed to have no effects on the overall expression level of TFIIH proteins (Supplementary Fig. S4(A,B)). Those results suggest TIE-1 increased NER activity.

**Figure 5:**
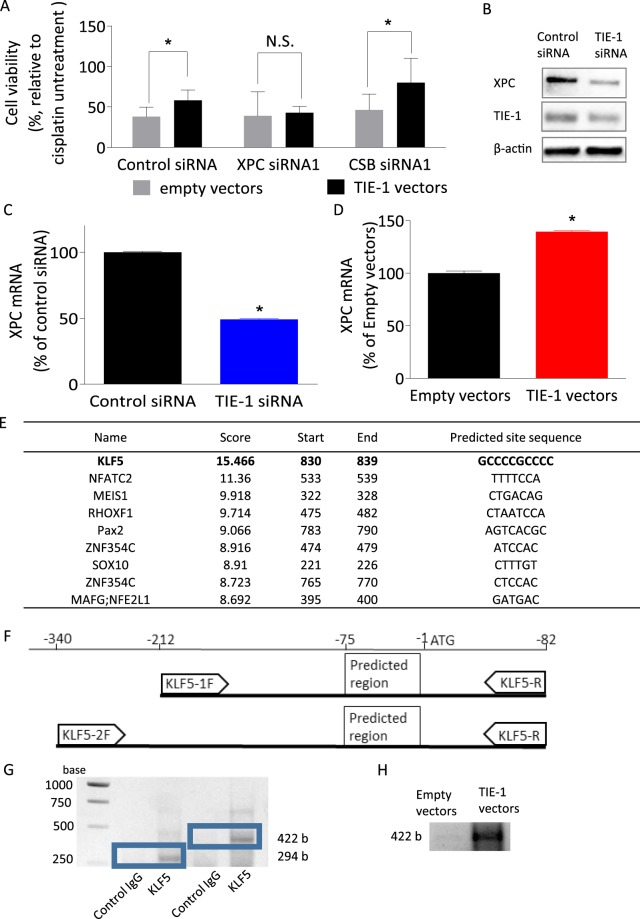
TIE-1 activates NER by increasing XPC. (**A**) TOV112D cells in 96-well plates were co-transfected with empty or TIE-1 vectors and siRNAs for XPC or CSB for 24 h and then treated with 3 μM cisplatin for 72 h. Cell viability was determined by Celltiter-Glo assay and expressed as the percentage relative to untreated cells. Values represent the mean ± SD of three independent experiments. (**B**) TOV112D cells were transfected with siRNAs or plasmid vectors for 24 h, followed by treatment with 10 µM cisplatin. Proteins and mRNAs were then extracted. Extracted proteins were submitted to immunoblot analysis using antibodies specific for XPC, TIE-1, and β-actin. Results are representative of three independent experiments. For the cropped blots, protein samples were run under same conditional treatments and processed in parallel. Full-length blots are presented in Supplementary Fig. S13. (**C**,**D**) XPC mRNA expression levels were determined by quantitative real-time PCR with a specific probe targeting XPC mRNA and normalized with glyceraldehyde 3-phosphate dehydrogenase mRNA expression levels. Values represent the mean ± SD of three independent experiments. (**E**) The XPC promoter region sequence was obtained from the NCBI online database and transcription-factor-binding sites in the XPC promoter region were predicted using the JASPAR database. (**F**) Designed PCR primers for ChIP assay. (**G**) Direct bindings of KLF5 to the promoter region of XPC in TOV112D cells were detected by PCR. (**H**) Direct binding of KLF5 to the promoter region of XPC was increased in TIE-1 overexpressed TOV112D cells. Three independent experiments were performed and representative picture was shown.

Xeroderma Pigmentosum Complementation Group C (XPC) and Cockayne syndrome group B protein (CSB) serve as key proteins in the damage-recognition step, responsible for global genomic and transcription-coupled NER, respectively^19^. Inhibition of XPC but not CSB by specific siRNAs attenuated cisplatin resistance induced by TIE-1 over-expression (Fig. 5(A), Supplementary Fig. S5(A,B)). Those results suggest that XPC-dependent NER is involved in TIE-1-driven cisplatin-resistance.

To define the molecular mechanisms whereby XPC mediates TIE-1-driven platinum-resistance, we investigated the effects of TIE-1 expressions on XPC transcription. TIE-1 knock-down significantly suppressed expression of XPC proteins and mRNAs (Fig. 5(B,C), Supplementary Fig. S6(A,B)), conversely TIE-1 over-expression increased XPC mRNA expression (Fig. 5(D)). Those results suggest that TIE-1 transcriptionally up-regulates XPC. Therefore, TIE-1-XPC pathway is suggested to increase NER activity.

To determine the transcription factors responsible for regulating XPC by TIE-1, an *in silico* search of XPC promoter regions using JASPAR (http://jaspar.genereg.net/) public database was performed. Krüppel-like factor 5 (KLF5) was identified as the most promising candidate transcription factor for XPC (Fig. 5(E)).

KLF5 was previously reported to up-regulate XPC expression and promote DNA repair^21^. We next investigated if KLF5 binds to the XPC promotor region. In chromatin immunoprecipitation (ChiP) assay, DNA fragments isolated by chromatin immunoprecipitation using KLF5 antibody were detected by PCR using primer pairs that are designed to amplify the region containing the KLF5 binding site (Fig. 5(F,G)). This result strongly suggests that KLF5 binds to XPC promoter region. Importantly, in TIE-1 overexpressing cells, we observed increased direct binding of KLF5 to the promoter region of XPC compared to empty vectors (Fig. 5(H)).

**Figure 6:**
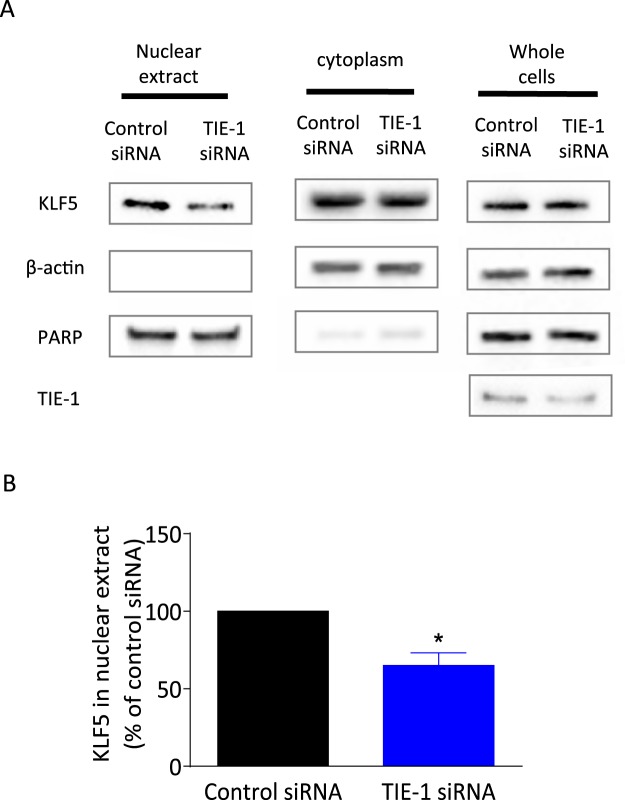
TIE-1 activates transcription factor KLF5. (**A**) TOV112D cells were transfected with control or TIE-1 siRNAs for 24 h. After harvesting cells, nuclear and cytoplasmic fractions were obtained by differential centrifugation. Results are representative of three independent experiments. For the cropped blots, protein samples were run under same conditional treatments and processed in parallel. Full-length blots are presented in Supplementary Fig. S14. (**B**) KLF5 levels in nuclear fraction were quantified and given as the mean ± SD of three independent experiments. *P < 0.05, **P < 0.01.

Activated KLF5 is re-localized to the nucleus where it acts as a transcription factor^22,23^. To determine if TIE-1 up-regulates KLF5, nuclear fractions were extracted from ovarian cancer cells to determine nuclear KLF5 protein levels. TIE-1 knock-down significantly led to decreases in nuclear KLF5 compared with control siRNA-treated cells without affecting whole cellular KLF5 levels (Fig. 6(A,B)), suggesting that TIE-1 promoted re-localization of KLF5.

Those results demonstrate that TIE-1 promotes KLF-dependent XPC expression.

### TIE-1 Inhibition Sensitizes Ovarian Cancer Cells Selectively to DNA damaging Anticancer Agents

**Figure 7:**
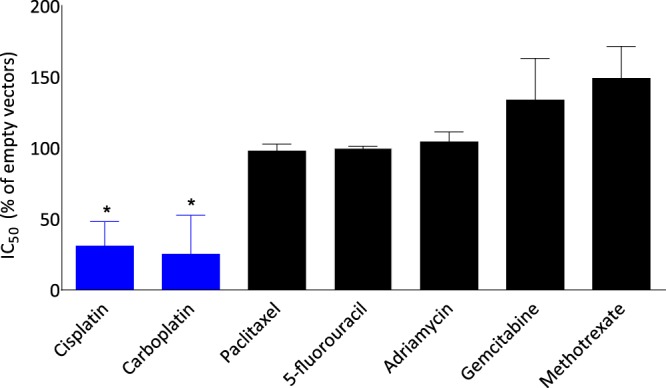
TIE-1 determines chemo-sensitivities to DNA-toxic anticancer agents. A2780CP cells were transfected with control or TIE-1 siRNAs for 24 h and then treated with various anticancer agents including gemcitabine, carboplatin, cisplatin, adriamycin, 5-fluorouracil, paclitaxel, and methotrexate for 72 h. Cell viability was assessed by Celltiter-Glo assay and IC_50_ values were determined. Values represent the mean ± SD of three independent experiments.

We investigated whether TIE-1 determines sensitivity to other chemotherapeutic agents as well as cisplatin. We evaluated the effects of TIE-1 knock-down on the sensitivities of ovarian cancer cells to seven chemotherapeutic agents, which have different mechanisms of cancer cell killing. In cisplatin-resistant A2780CP cells, TIE-1 inhibition with siRNA significantly decreased the IC_50_ values for cisplatin and carboplatin by 31% and 26%, respectively, while IC_50_ values for non-DNA damaging reagents such as paclitaxel, 5-fluorouracil, and adriamycin remained unchanged (Fig. 7). Surprisingly, the IC_50_ values for gemcitabine and methotrexate were increased by 37% and 51%, respectively. In cisplatin-sensitive TOV112D cells, TIE-1 overexpression increased the IC_50_ values of cisplatin and carboplatin (Supplementary Fig. S8). Those results suggest that TIE-1 significantly affected the sensitivity to DNA-adduct-type chemotherapeutic reagents such as cisplatin and carboplatin.

Overall, our results suggest that TIE-1 increases NER and DNA repair by up-regulating transcription of XPC, thereby rendering ovarian cancer cells resistant to DNA-adduct-type chemotherapeutic reagents.

## Discussion

Fully characterizing molecular mechanisms of chemo-resistance is critical for drug discovery. Herein we demonstrated that an orphan receptor tyrosine kinase TIE-1 up-regulates XPC-dependent NER rendering ovarian cancer cells resistant to platinum reagents and is a key determinant responsible for the therapeutic efficacy of DNA-damaging reagents in ovarian cancer by controlling a DNA repair system. Importantly TIE-1 is proposed to represent a novel therapeutic target for potentiating chemotherapeutic efficacy in ovarian cancer, especially in patients with platinum-resistant cancer.

Numerous genes have been implicated in platinum resistance, such as multidrug resistance protein 2 (MRP2) involved in cisplatin efflux^7^, glutathione and metallothionein involved in platinum detoxification^8^, and excision repair cross-complementation group 1 (ERCC1) involved in DNA damage repair^10^. In this study, TIE1 emerged as a novel sensitizer among other families of genes already shown to affect cisplatin sensitivity.

TIE-1 is known to play a critical role in angiogenesis and blood-vessel stability by inhibiting angiopoietin-1 signaling through the endothelial receptor tyrosine kinase receptor TIE-2^24,25^. These two receptors have remarkably similar structures including two amino-terminal immunoglobulin domains followed by three epidermal growth factor repeats, a third immunoglobulin domain, and finally, three fibronectin type-III repeats in the extracellular region. Both also contain a catalytic carboxyl-terminal tyrosine kinase domain responsible for intracellular signaling. TIE-1 cooperates with TIE-2 by forming the hetero complex during angiopoietin signaling^26^. Angiopoietins and the TIE-2 receptor are considered as important regulators of tumor-induced angiogenesis, cancer-cell growth, and metastasis^27,28^. These facts raise the question whether effects of TIE-1 knock-down or over-expression on cisplatin sensitivity might be mediated through TIE-2 receptor signaling. In our consideration, however, the effects of TIE-1 observed in our study are independent with TIE-2 action for several reasons. First, TIE-2 was included in the siRNA library used for the primary screening and it was not identified as a hit. Second, TIE-2 knock-down had no effect on cisplatin sensitivity of ovarian cancer cells (Supplementary Fig. S7(A)). Third, Kaplan-Meier analysis of a public database showed that high TIE-2 expression was not correlated with progression free survival in ovarian cancer patients (Supplementary Fig. S7(B)). These results indicate that TIE-1 is involved in rendering ovarian cancer cells resistant to cisplatin independently of TIE-2.

Over-expression of TIE-1 has been reported in gastric cancer^29^, colon cancer^30^, and breast cancer^31^ cells, but the significance of this is not clear. Our studies point to potential clinical significance of TIE-1 in ovarian cancer pathobiology. A public database analysis showed that high TIE-1 expression was correlated with a poor prognosis in ovarian cancer patients. More importantly, we found higher expression of TIE-1 in refractory stage III or IV ovarian cancer patients who had residual tumor on first surgery. Our data suggest that TIE-1 could be related to patients’ prognosis in clinical practice by regulating platinum-sensitivity.

Kontos *et al*. proposed that TIE-1 activates the phosphatidylinositol 3-kinase (PI3K)-AKT pathway and inhibits UV-induced apoptosis^32^. TIE-1 might promote cell survival by PI3K-AKT pathway activation. PI3K and its subunits (mainly PIK3CA) are known for being highly mutated and responsible for increasing chemo-resistance in ovarian cancer^33,34^. Downstream of PI3K, AKT isoforms (AKT1-2-3) have been reported to also increase chemo-resistance against platinum drugs^34^. In our study, the PI3K-AKT pathway could contribute to TIE-1-induced chemo-resistance but further investigation will be required.

Genetic approaches revealed that TIE-1 up-regulates KLF5-XPC-dependent NER. Zhang *et al*.^35^ reported that phosphorylation of KLF5 at the cAMP-response element-binding protein interaction domain enhanced its transactivation function. We observed significant TIE-1-dependent translocation of KLF5 to the nuclear fraction, however KLF5 phosphorylation was not confirmed because specific antibodies were unavailable. As the molecular mechanisms by which TIE-1 up-regulates KLF5 thus remain unknown, further investigations are required to characterize the novel TIE-1-KLF-5 pathway in XPC-dependent NER and to improve our understanding of its pathobiological significance.

**Figure 8:**
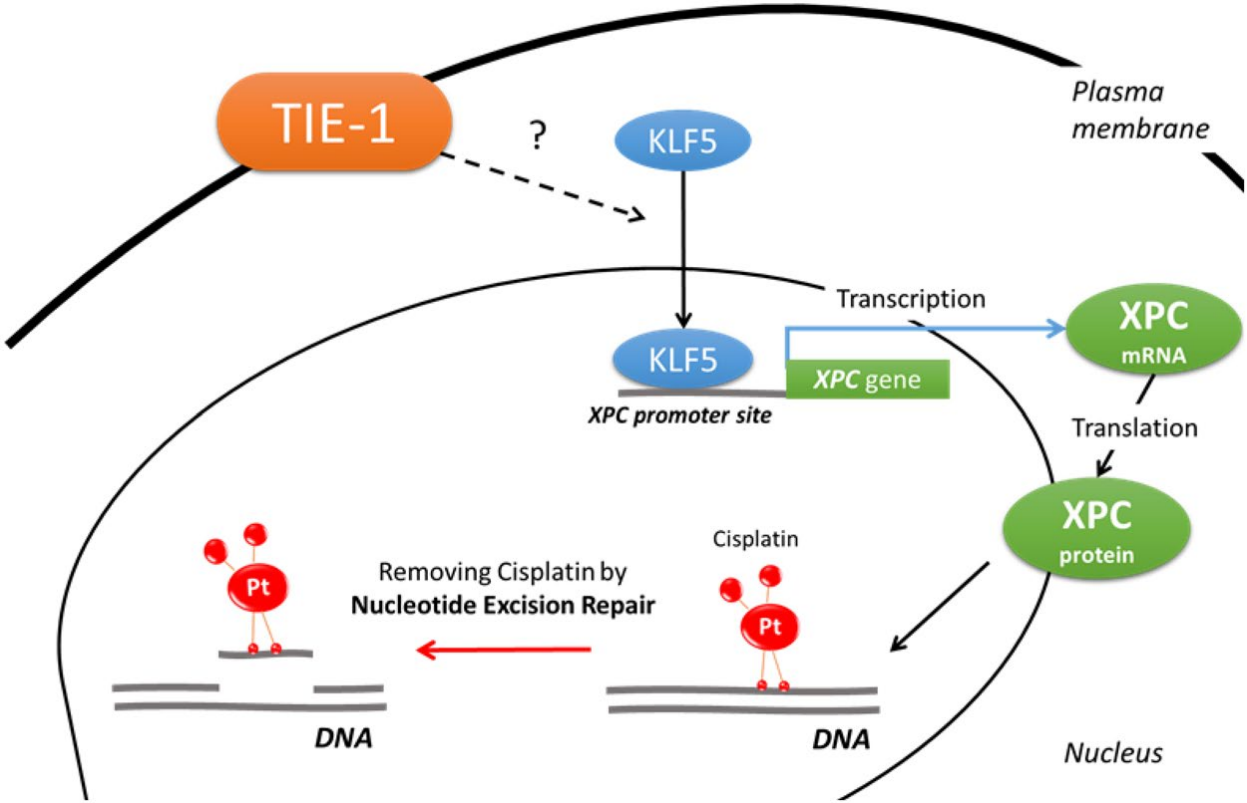
Schematic diagram illustrating TIE-1 involvement in decreased susceptibility to DNA damage by cisplatin. Pt: platinum.

In summary, we demonstrated that TIE-1 is a key molecule involved in platinum resistance of ovarian cancer cells. This platinum resistance is mediated by the transcription factor KLF5, which in turn promotes XPC expression and subsequent NER and DNA repair (Fig. 8). We therefore propose that TIE-1 represents a promising therapeutic target for potentiating chemotherapeutic efficacy in patients with ovarian cancer. Moreover, our studies possibly promote the identification of TIE-1 ligands in the future.

## Materials and Methods

### Reagents

Antibodies specific for TIE-1 (c-18) and TFIIH were purchased from Santa Cruz Biotechnology (Dallas, TX, USA). KLF5 antibody was purchased from Abcam (Cambridge, MA, USA). Antibodies specific for XPC, cleaved-PARP, phospho-histone H2A.X (ser 139), and H2A.X were purchased from Cell Signaling Technology (Boston, MA, USA). Cc3-conjugated V5 antibody and β-actin antibody (A5441) were from Sigma (St. Louis, MO, USA). Hoechst 33342 was obtained from Dojindo (Kumamoto, Japan). Cisplatin, adriamycin, paclitaxel, 5-fluorouracil, methotrexate, carboplatin, and gemcitabine were purchased from Wako (Japan). Human TIE-1 cDNA was purchased from Kazusa DNA Research Institute (Kisarazu, Japan). Ovarian cancer cell lines were from the American Type Culture Collection. SiRNAs of TIE-1 (s14140, s14141), TIE-2 (s13983), XPC (s14929, s14930) and CSB (s 4806, s4807) are from Thermo fisher Scientific (Waltham, MA U.S.A.).

### Cell culture

Ovarian cancer cell lines (A2780, A2780CP, IGROV1, IGROVCP, PE01, PE04, and TOV112D) were cultured in Dulbecco’s modified Eagle’s medium (DMEM) supplemented with 10% fetal bovine serum. A2780CP and IGROVCP were established from their parental cells A2780 ovarian serous adenocarcinoma cell and IGROV1 ovarian endometrioid adenocarcinoma cell *in vitro*, respectively^36,37^. PE01 and PE04 cells were adenocarcinoma cell lines independently established from a single patient pre- and post-cisplatin-resistance acquisition, respectively^38^. TOV112D is ovarian endometrioid adenocarcinoma cell line^39^.

### High-throughput siRNA screening

Genome-wide siRNA screens were performed in 384-well plates using robotic instrumentation at the Quellos High Throughput Screening Facility at the University of Washington’s Institute for Stem Cells and Regenerative Medicine (Seattle, WA). The siRNA library targeted about 6659 unique human genes, with three siRNAs *per* gene, the sequences of which were designed using an algorithm developed to increase efficiency of the siRNAs for silencing, while minimizing their off-target effects. The screen was carried out in triplicate, with viability as the phenotypic endpoint. A2780CP ovarian cancer cells were plated in 384-well plates in 50 µl per well of complete medium using a WellMate (Matrix Technologies, Canada) and transfected with siRNAs 24 h later using Lipofectamine RNAi MAX Reagent (ThermoFisher Scientific, MA, USA), with three siRNAs targeting the same gene pooled at equal molarities (final concentration of each siRNA, 5 nM). Cells were treated with cisplatin 0.01 µM (IC_20_) or vehicle at 24 h following transfection, and the plates were incubated at 37 °C in a 5% CO_2_ incubator for 72 h. Cell viability was assessed by CellTiter-Glo assay (Promega, WI, USA), and chemiluminescence was quantified using an Envision multilabel plate reader (PerkinElmer Life Sciences, MA, USA). Raw luminescence values were mock normalized per plate and plotted for distribution and datamining (Miner 3D software, Miner3D, DE, USA) using a negative control siRNA (siLuc) and a positive control highly toxic siRNA targeting the kinesin motor protein Kif11. The criteria for selecting hit 320 genes are shown in Supplementary Table 2.

### Validation of 30 potential gene hits

Thirty cisplatin-sensitizing hits in the high-throughput screening were validated with newly synthesized specific siRNAs. A2780CP and PE04 cells plated in 96-well plates were transfected with control or individual-gene siRNAs (5 nM) for 24 h. After transfection, cells were treated with cisplatin 3 μM or vehicle for 72 h and cell viability was assessed by Celltiter-Glo and luminescence analysis.

### Human tissue samples

Thirteen human tissue samples were obtained from the Surgical Pathology Archives of the Obstetrics and Gynecology Department of Tohoku University Hospital 2007–2016 (Sendai, Japan). Samples were obtained from advanced (clinical stage III or IV) ovarian cancer patients who underwent primary surgery, and had six cycles of chemotherapy with carboplatin and paclitaxel. Patients who were not observed to progress clinically within the period of chemotherapy were considered as platinum-sensitive, and patients who were observed to progress within the period of chemotherapy were considered as platinum-resistant. Tissues from five platinum-sensitive and eight -resistant patients were evaluated. Tissues were fixed in buffered-formalin and embedded in paraffin, followed by staining with hematoxylin and eosin or TIE-1 antibody. This protocol was approved by the Ethics Committee of Tohoku University Graduate School of Medicine (Sendai, Japan). All methods were performed in accordance with the protocol and written informed consent was obtained from the patients. Patient characteristics are described in Supplementary Table 1.

### Immunohistochemistry

TIE-1 immunohistochemistry was performed with the modified methods^40^. After deparaffinization, tumor tissues were stained with anti-TIE-1 (ab11547, Abcam) antibody (Abcam, UK). Immunohistochemical analysis was performed by the streptavidin–biotin amplification method using a Histofine kit (Nichirei, Japan) and immunohistochemical staining of tumor tissues for TIE-1 was evaluated. Vascular endothelial cells were used as positive controls. Cells that were stained with TIE-1 antibody more strongly than vascular endothelial cells were determined as “TIE-1 positive”, and cells that stained the same or weaker than vascular endothelial cells were determined as “TIE-1 negative”. Immuno-stained sections were evaluated by three independent, blinded observers.

### Preparation of TIE-1 vectors

TIE-1 cDNA was amplified by polymerase chain reaction (PCR) using the sets of primers, TIE-1-1FstartpstEcoRI: 5′-ctgcaggaattcatggtctggcgggtg-3′ containing an EcoRI site, and TIE-1-3414RnonsopkpnXbaI: 5′-ggtacctctagaggcctcctcagctgt-3′ containing an XbaI site without a stop codon. The PCR product and pcDNA3.1/V5-HisA vector were digested with EcoRI/XbaI and the fragments were ligated with T4 DNA Ligase (TaKaRa, Japan). The sequence of the produced plasmid was verified with T7 and BGH primers.

### Transfection with TIE-1 vectors

Ovarian cancer cells were transfected with the vectors using Lipofectamine 2000 according to the manufacturer’s instructions.

### Immunofluorescence

Indirect immunofluorescence microscopy was performed with the modified method^41^. Briefly, cells plated on glass-bottomed 35 mm dishes were washed with phosphate-buffered saline (PBS), fixed with 4% formaldehyde in PBS for 10 min at room temperature, and then treated with 0.1% Triton-X100 for 10 min. Cells were blocked with 20% human serum in PBS for 1 h followed by treatment with primary antibodies in PBS containing 20% human serum at 4 °C overnight. Cells were washed with PBS, treated with Alexa488- and/or Alexa555-conjugated anti-IgG antibodies in PBS containing 20% human serum for 1 h, and then stained with Hoechst 33342. Confocal laser microscopy was performed using an LSM780 confocal microscope (Carl Zeiss, Thornwood, NY, USA).

### Western blotting

Cells were washed with ice-cold PBS three times and harvested cells were lysed using Laemmli buffer. Cellular proteins were subjected to sodium dodecyl sulfate-polyacrylamide gel electrophoresis in a 4–20% gradient gel. Proteins were electrophoretically transferred to nitrocellulose membranes and then blocked with PBS/0.1% Tween 20 (PBS-T) containing 5% nonfat dried milk. Membranes were incubated with primary antibodies at 4 °C overnight. The blots were washed with PBS-T and incubated with horseradish peroxidase-conjugated secondary antibody in PBS-T containing 5% nonfat dried milk. The proteins were detected using SuperSignal West Dura Extended Duration Substrate and ChemiDoc™ MP (Bio-Rad).

### Platinum determination by AAS

After treatment with cisplatin, cells were washed with PBS three times and then lysed with 0.1 M NaOH. AAS was carried out with an iCE3500 spectrometer with graphite furnace (Thermo Scientific, Cambridge, UK). The spectrometer was controlled by specific software SOLAAR (Thermo Scientific, version 11.02). The selected wavelength of platinum was 265.9 nm. Background collection was performed by the deuterium lamp method. The heating programs are shown in Supplementary Table 3. The SOLAAR software package was also used for data processing.

### Quantitative real-time PCR

Cell homogenization and RNA extraction were performed using the QIAshredder and RNeasy mini kits (Qiagen, Hilden, Germany) according to the manufacturer’s manual. Extracted RNAs (1 µg) were reverse transcribed to generate cDNAs using SuperScript III (Thermo Fisher Scientific). Real-time PCR for determining TIE-1 and XPC mRNAs was performed using the StepOne Plus Real-Time PCR System with TaqMan Universal Master Mix II and a TaqMan probe specific for each gene (Thermo Fisher Scientific).

### UV irradiation

Cells were seeded into 35 mm glass-bottomed dishes at 2 × 10^5^/well (TOV112D, A2780CP) and transfected empty vectors or TIE-1 vectors. Following 24 hour after transfection, cells were treated with UVC (254 nm) from a UV Stratalinker 1800 (Stratagene). Five minutes after UVC treatment, cells were fixed and stained by TFIIH antibody.

### ChIP assay

The ChIP assay was performed according to the manual (Cosmobio, Japan). Briefly, TOV112D cells were fixed with 1% formaldehyde. Covaris S220 Ultrasonicator (Covaris, Inc., USA) was used for sonicating to lysate to shear DNA to an average fragment size of 200–1000 bp. The size of DNA fragments was by Agilent 4200 Tapestation (Agilent Technologies, USA). The sonicated nuclear lysates were immunoprecipitated with anti-KLF antibody or rabbit IgG. After purification of the immunoprecipitated DNA, 422-bp and 294-bp region of the XPC promoter was amplified by PCR using the following primers: KLF5-1F: 5′-caccggaaatagagaaacctg-3′, KLF5-2F: 5′-cgagcgacctccttaaaatacac-3′, KLF5-R: 5′-ccggaacgagaaccggaac-3′ (NIPPON Genetics Co.Ltd, Japan).

### Nuclear extraction

Nuclear extraction was performed as described previously, with some modifications^42^. Cells were lysed in hypotonic buffer containing 10 mM HEPES (pH 7.9), 10 mM KCl, 0.2 mM EDTA, 0.5% Nonidet P-40, 1 mM dithiothreitol, and 0.5 mM phenylmethylsulfonyl fluoride. The lysates were centrifuged at 1,000 *g* for 3 min to sediment the nuclei, and the supernatant was assayed as cytosol. To extract proteins, the nuclear fraction was further incubated for 15 min in ice-cold hypertonic buffer containing 20 mM HEPES (pH 7.9), 400 mM NaCl, 2 mM EDTA, 1 mM dithiothreitol, and 1 mM phenylmethylsulfonyl fluoride.

### Statistical analysis

Values are presented as mean ± standard error of the mean based on at least three independent experiments. Images are representative of at least three independent experiments. Statistical analyses were performed using GraphPad Prism 6. Statistical significance was assessed by performing individual *t-*tests. *P* values < 0.05 were considered significant.

## Electronic supplementary material

Supplementary Information
