# Supplementary material for: Tyrosine kinase receptor TIE-1 mediates platinum resistance by promoting nucleotide excision repair in ovarian cancer
Source: Sci Rep. 2018 Sep 4;8:13207. doi: 10.1038/s41598-018-31069-2 (PMC6123490; doi:10.1038/s41598-018-31069-2)
Supplement: Supplementary file 1 — Supplementary Information [file 41598_2018_31069_MOESM1_ESM.pdf]

Supplementary information:

Tyrosine kinase receptor TIE-1 mediates platinum resistance by promoting nucleotide excision repair in ovarian cancer

Short title: Involvement of tyrosine kinase receptor TIE-1 in platinum resistance.

Masumi Ishibashi[a], Masafumi Toyoshima[a,*], Xuewei Zhang[a], Junko Hasegawa-Minato[a], Shogo Shigeta[a,c], Toshinori Usui[b], Christopher J. Kemp[c], Carla Grandori[d], Kazuyuki Kitatani[a,b,*], Nobuo Yaegashi[a]

a Department of Obstetrics and Gynecology, Tohoku University Graduate School of Medicine, Sendai, Japan
b Tohoku Medical Megabank Organization, Tohoku University Graduate School of Medicine, Sendai, Japan
c Division of Human Biology, Fred Hutchinson Cancer Research Center, WA, USA
d SEngine Precision Medicine, WA, USA

*Correspondence: Masafumi Toyoshima and Kazuyuki Kitatani

Masafumi Toyoshima, M.D., Ph.D., Department of Obstetrics and Gynecology, Tohoku University Graduate School of Medicine, 1-1 Seiryo-machi, Aoba-ku, Sendai, Miyagi, 980-8574, Japan.
Tel: +81-22-717-7251; Fax +81-22-717-7258; E-mail: m-toyo@tohoku.med.ac.jp

Kazuyuki Kitatani, Ph.D., Tohoku Medical Megabank Organization, Tohoku University Graduate School of Medicine, 2-1 Seiryo-machi, Aoba-ku, Sendai, Miyagi, 980-8573, Japan.
Tel: +81-022-717-7251; Fax +81-22-717-7258; E-mail: kitatani@med.tohoku.ac.jp

Supplementary Figures:

Supplementary Figure S1: Overall survival of TIE-1 high cases treated with platinum.

A data set of 1409 normalized ovarian cancer microarrays treated with platinum drugs was downloaded from the Kaplan–Meier Plotter database and overall survival curves for high expression (defined as cancers with values above the median) or low expression of TIE-1 were analyzed.

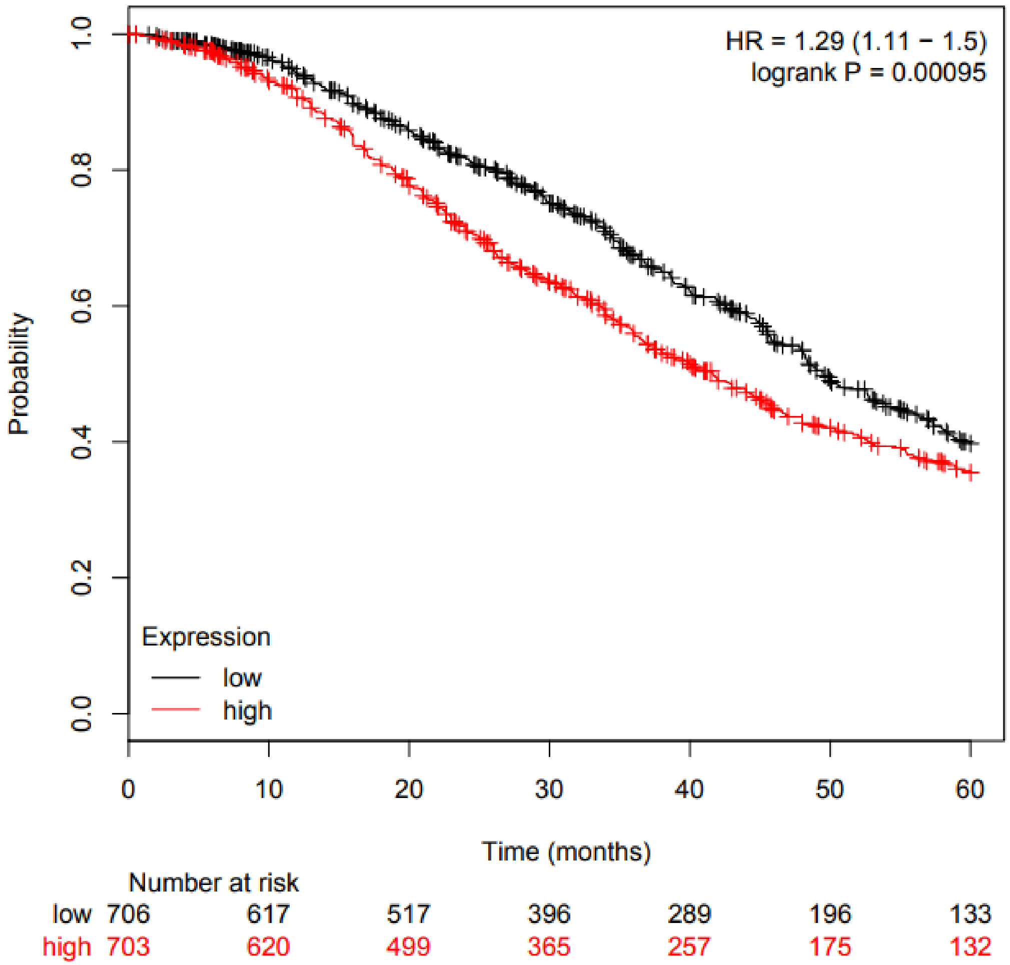

Supplementary Figure S2: TIE-1 expression correlates cisplatin sensitivity .

Scattered plot shows that TIE-1 protein expression and IC_{50} of each cell lines were correlate. The value for the correlation coefficient were 0.5268.

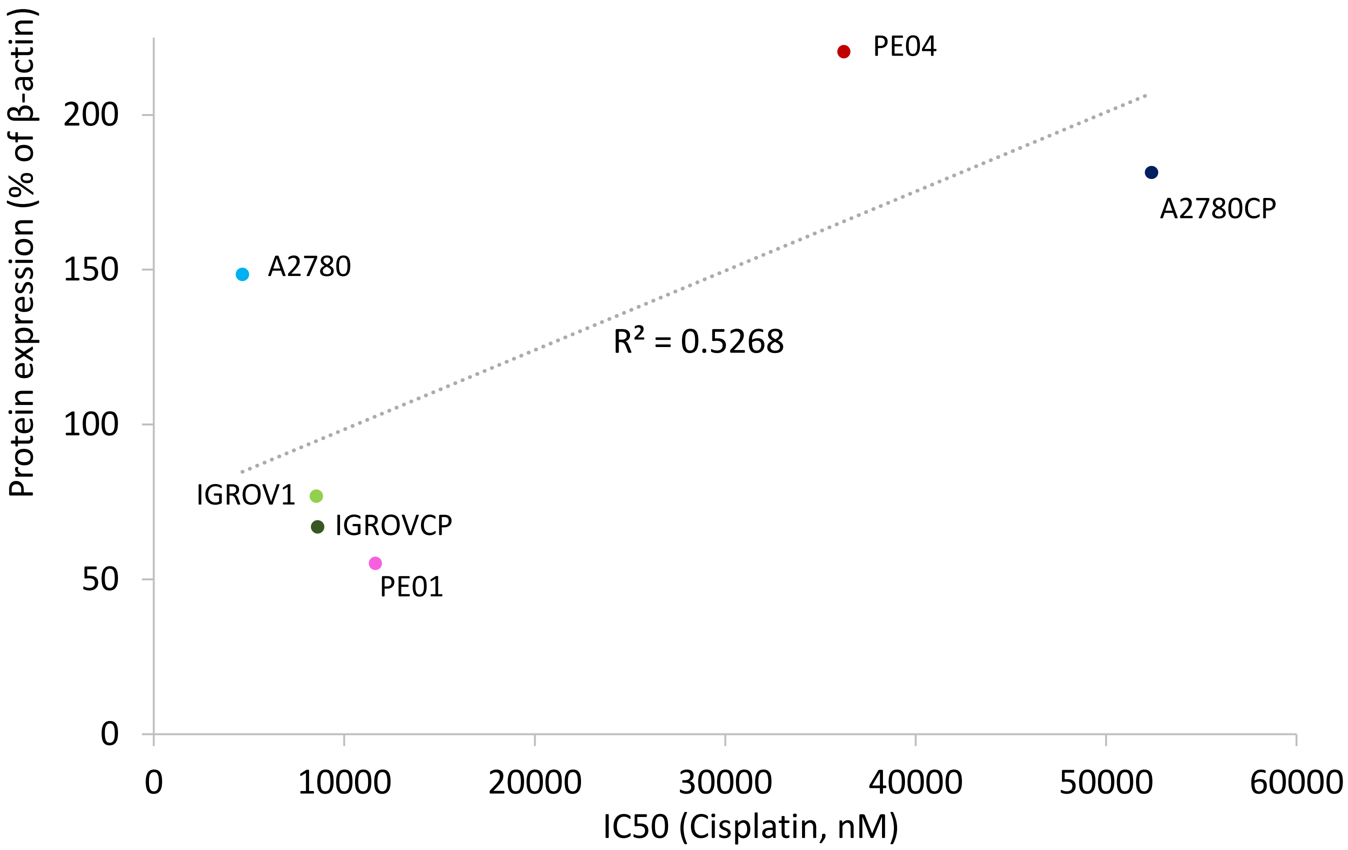

Cell lines	IC_{50}(nM)
A2780	4650
A2780CP	52379
PE01	11630
PE04	36228
IGROV1	8528
IGROVCP	8603

Supplementary Figure S3: Kinetics of γH2AX .

After cisplatin (3μM) treatment, γH2AX was detected in immunofluorescence. TOV112D cells were examined.

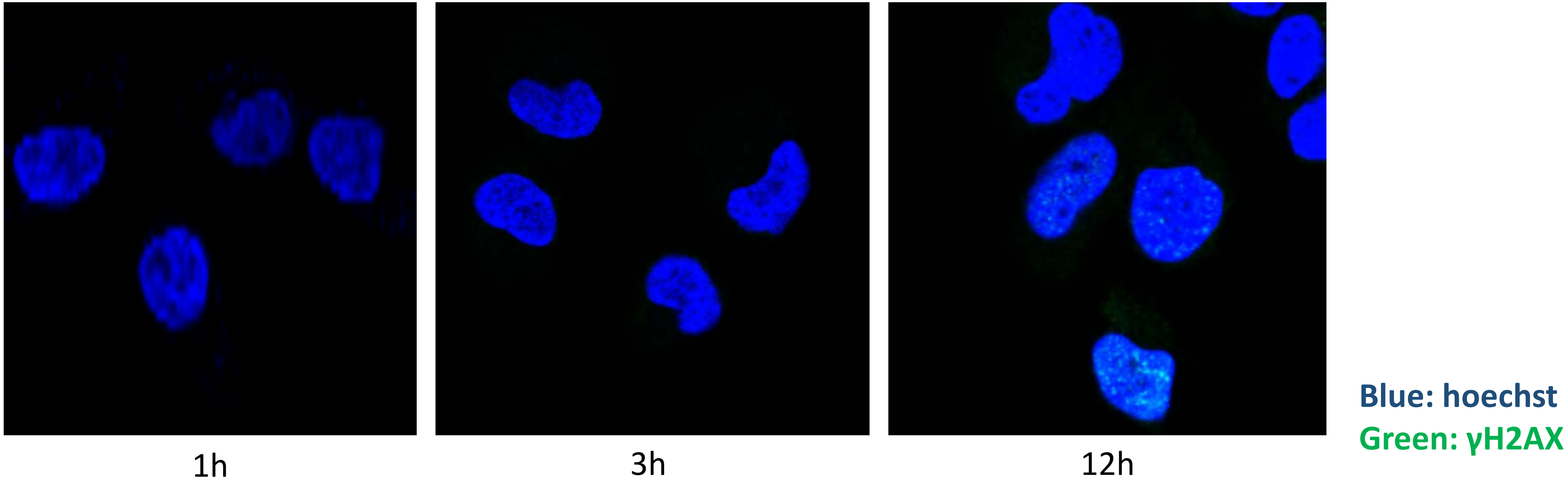

Supplementary Figure 4: TFIIH protein expression did not change by TIE-1 knockdown.

A, A2780CP cells were transfected with the control#2 siRNAs and TIE-1 siRNAs (5 nM) for 24h. After transfection, cells were treated with 10 μM cisplatin, and then proteins were extracted. Extracted proteins were submitted to immunoblot analysis using antibodies specific for TFIIH. The expression of TFIIH was determined by immunoblotting. B, TFIIH protein expression levels were quantified values represent the mean ± SD of three independent experiments.

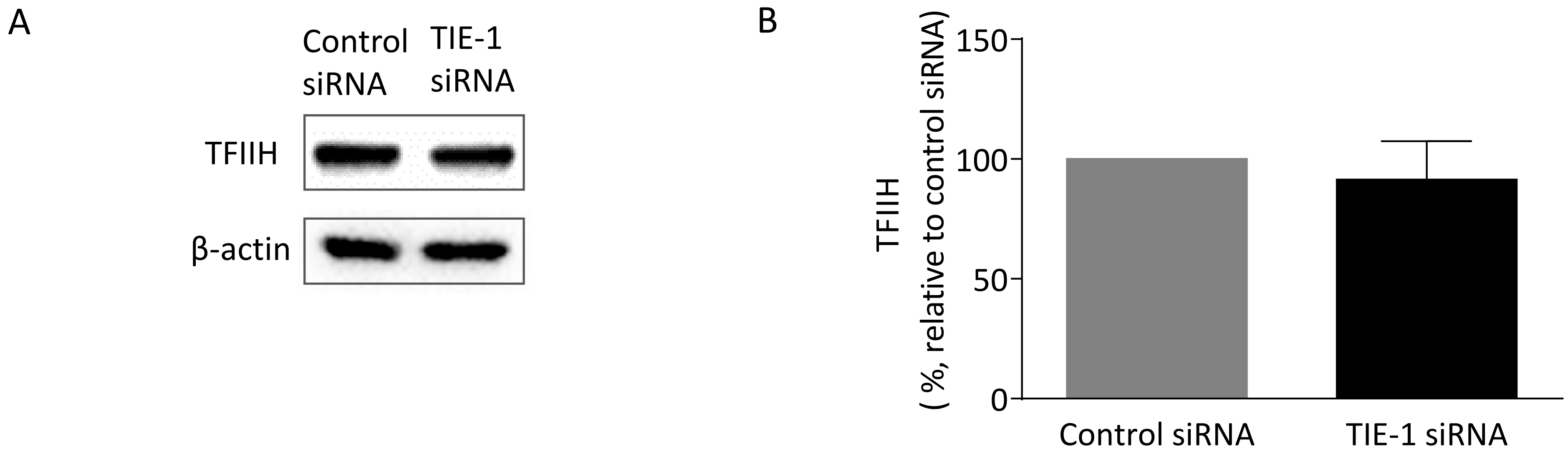

Supplementary Figure S5: XPC and CSB knock-down by different sequence siRNAs .

A, TOV112D cells in 96-well plates were co-transfected with empty or TIE-1 vectors and different sequence siRNAs for XPC or CSB for 24 h and then treated with 3μM cisplatin for 72 h. Cell viability was determined by Celltiter-Glo assay and expressed as the percentage relative to untreated cells. Values represent the mean ± SD of three independent experiments. B, XPC and CSB mRNA levels were quantified values represent the mean ± SD of three independent experiments.*: $p < 0.05$

A

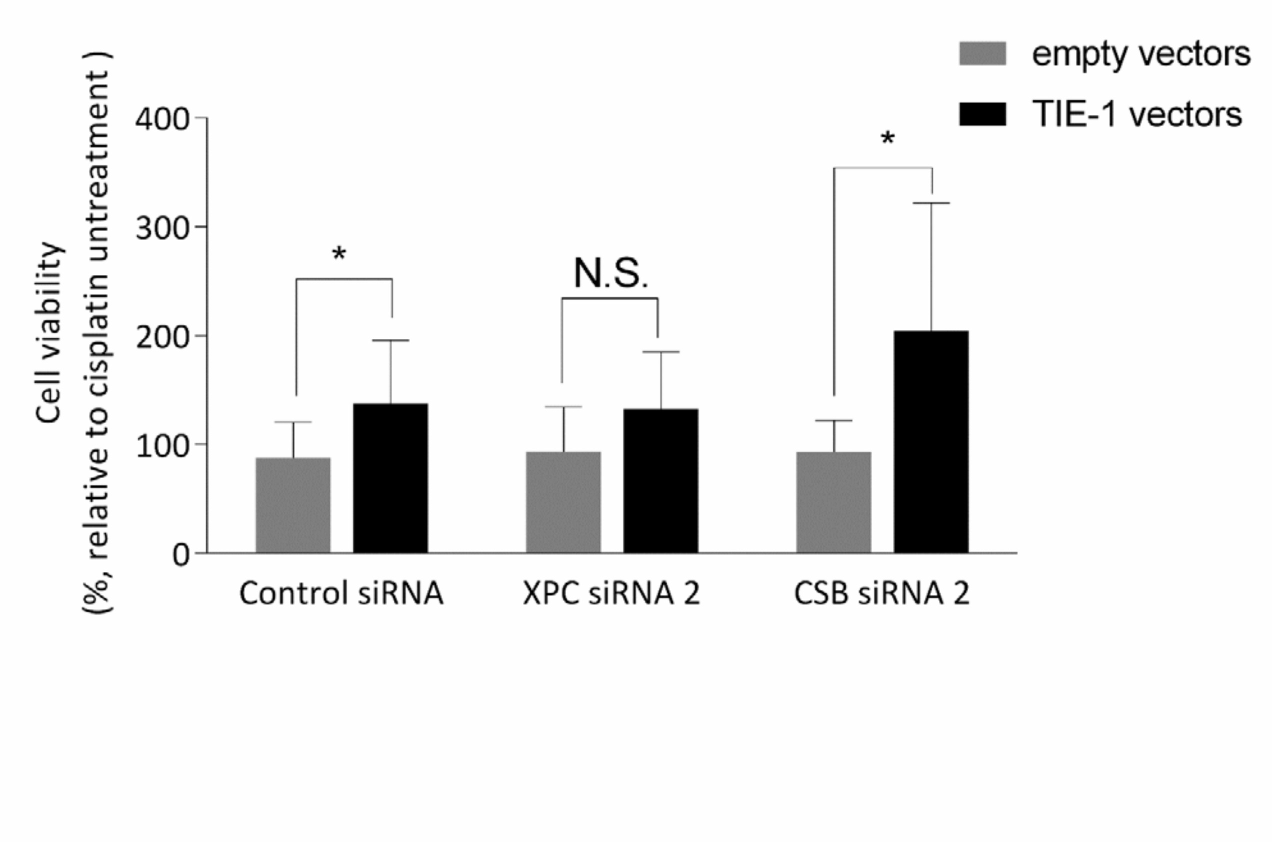

B

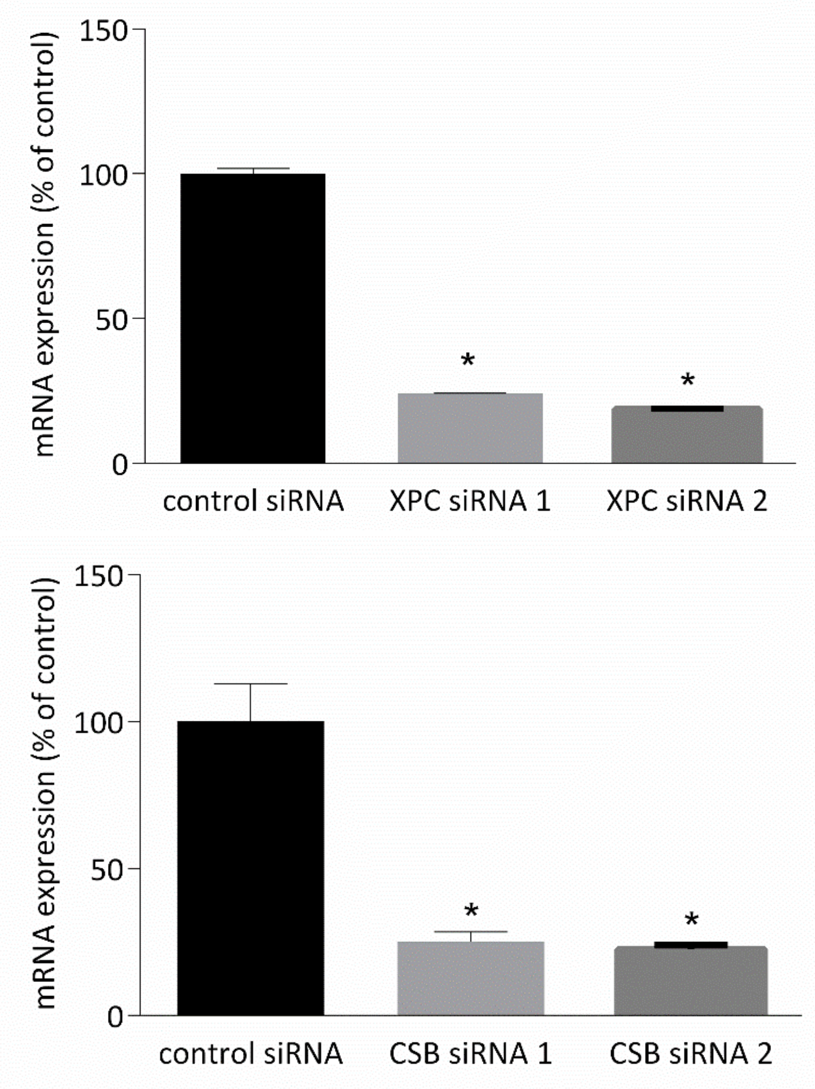

Supplementary Figure S6: TIE-1 knock-down by different sequence TIE-1 siRNA .

A, TIE-1 mRNA levels were quantified values represent the mean ± SD of three independent experiments. B, XPC mRNA levels were quantified values represent the mean ± SD of three independent experiments.

*: $p < 0.05$, **: $p < 0.01$

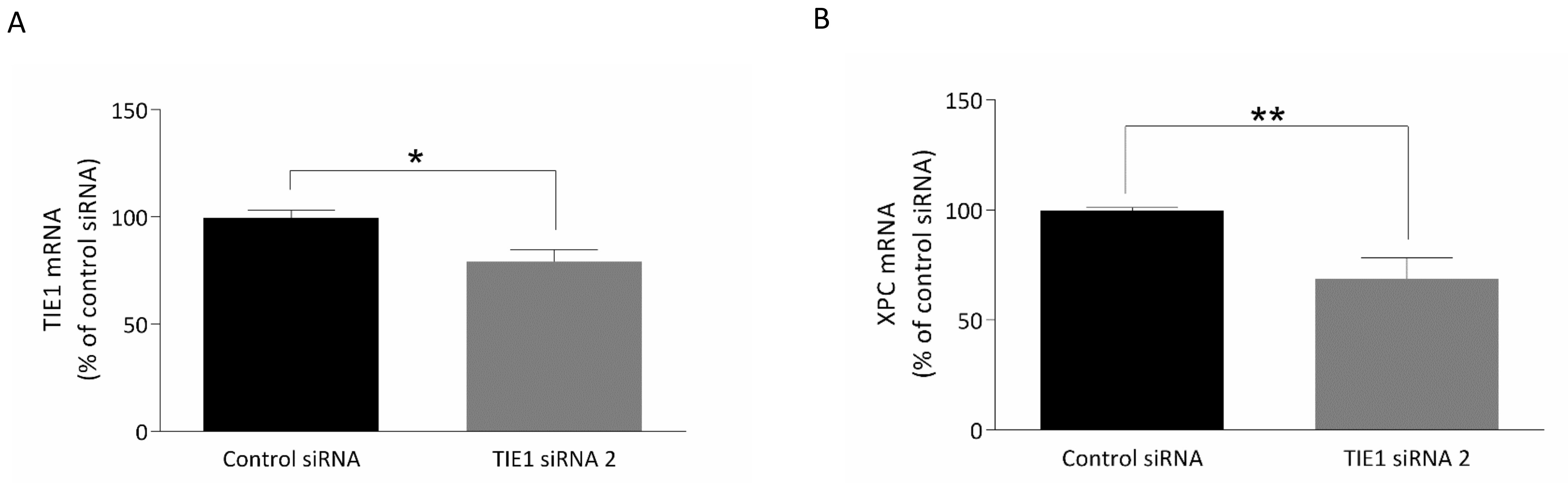

Supplementary Figure S7: TIE-1 is involved in transforming to cisplatin resistant independently of TIE-2.
A, TOV112D cells plated in 96-well plates were transfected with control#2 siRNAs or TIE-1 siRNAs or TIE-2 siRNAs (5 μM) for 24 hours. After transfection, cells were treated with cisplatin (0, 0.3, 1, 3, 10, 30, 100 μM) for 72 hours and then cell viability was determined by Celltiter-Glo assay. The IC_{50} values represent mean ± SD of three independent experiments. B, A data set of 1656 normalized ovarian cancer microarrays was downloaded from the Kaplan–Meier Plotter database and overall survival curves for high expression (defined as cancers with values above the median) or low expression of TIE-2 were analyzed.

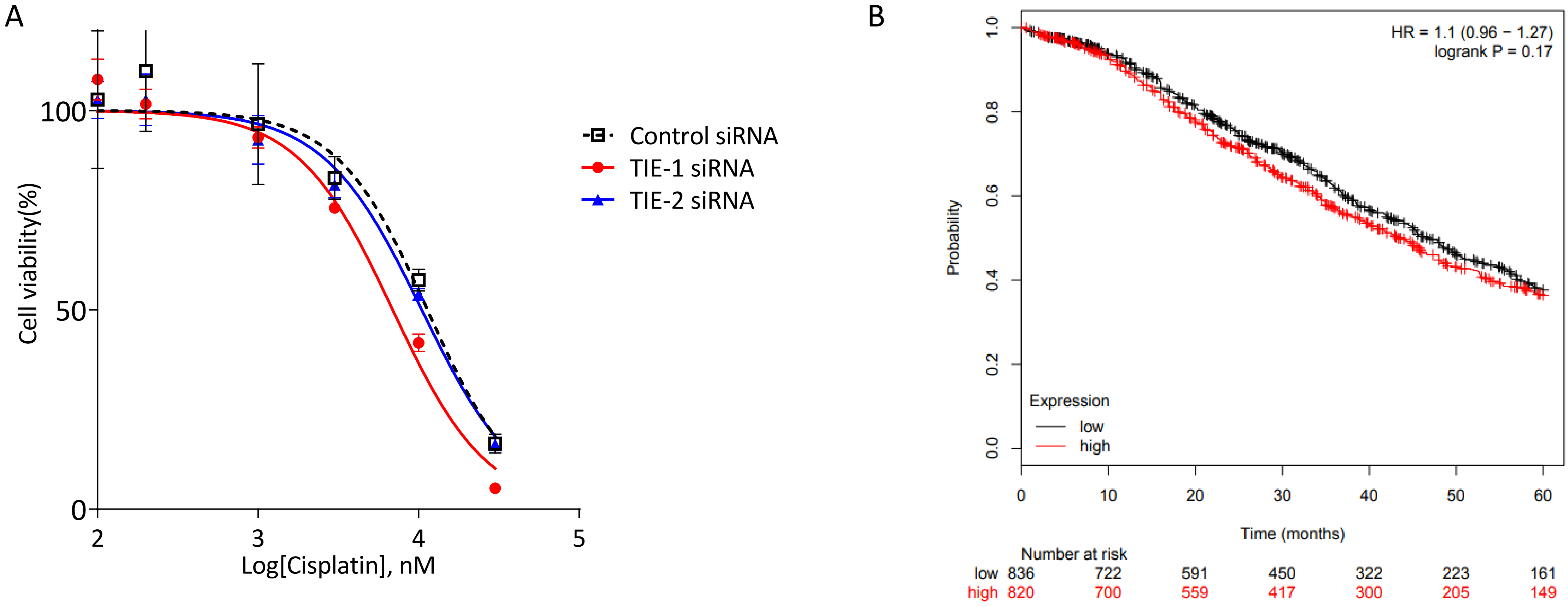

Supplementary Figure S8. TIE-1 determines chemo-sensitivities to DNA-toxic anticancer agents. TOV112D cells were transfected with empty vectors or TIE-1 vectors for 24 h and then treated with various anticancer agents including gemcitabine, carboplatin, cisplatin, adriamycin, 5-fluorouracil, paclitaxel, and methotrexate for 72 h. Cell viability was assessed by Celltiter-Glo and IC_{50} values were determined. Values represent the mean ± SD of three independent experiments. *$p < 0.05$

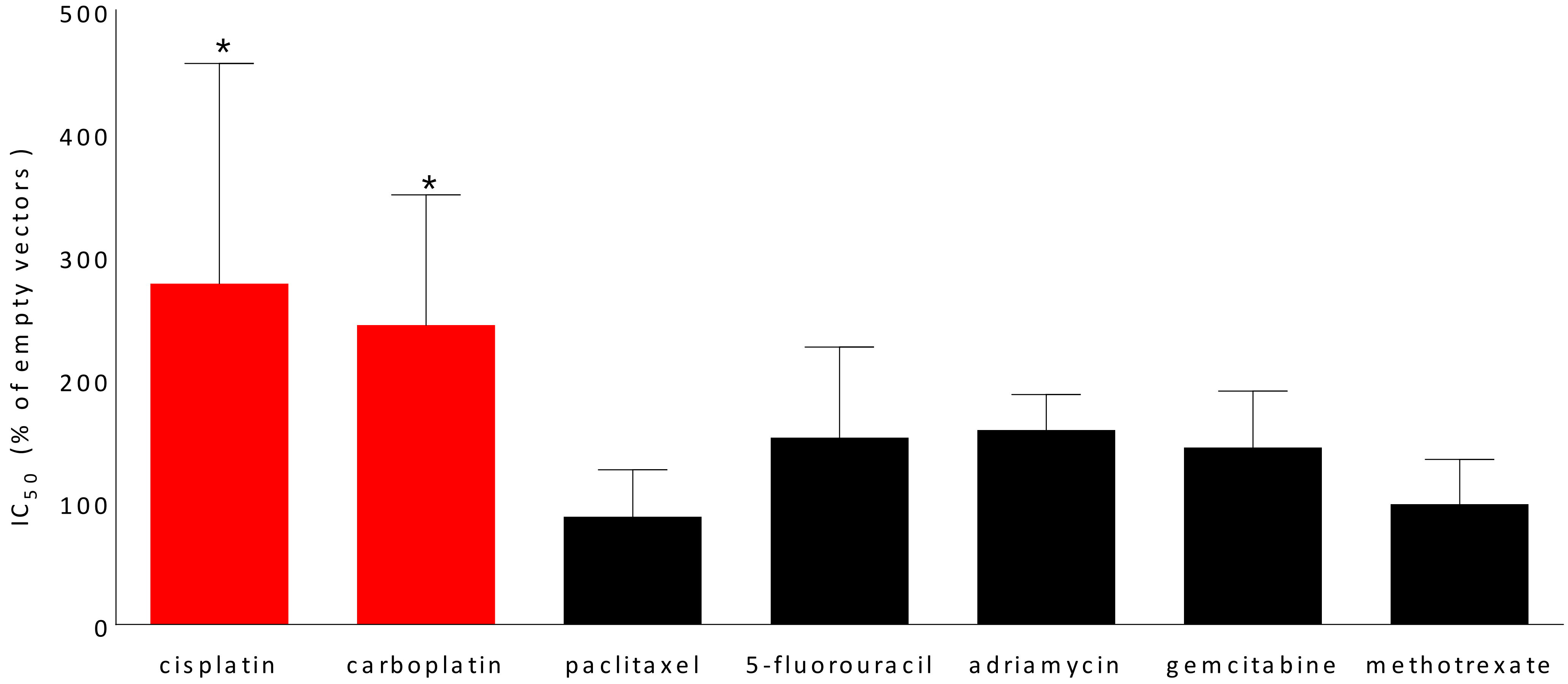

Supplementary Figure S9. Full-length blots representing the protein expressions of TIE-1 as shown in Figure 1c.

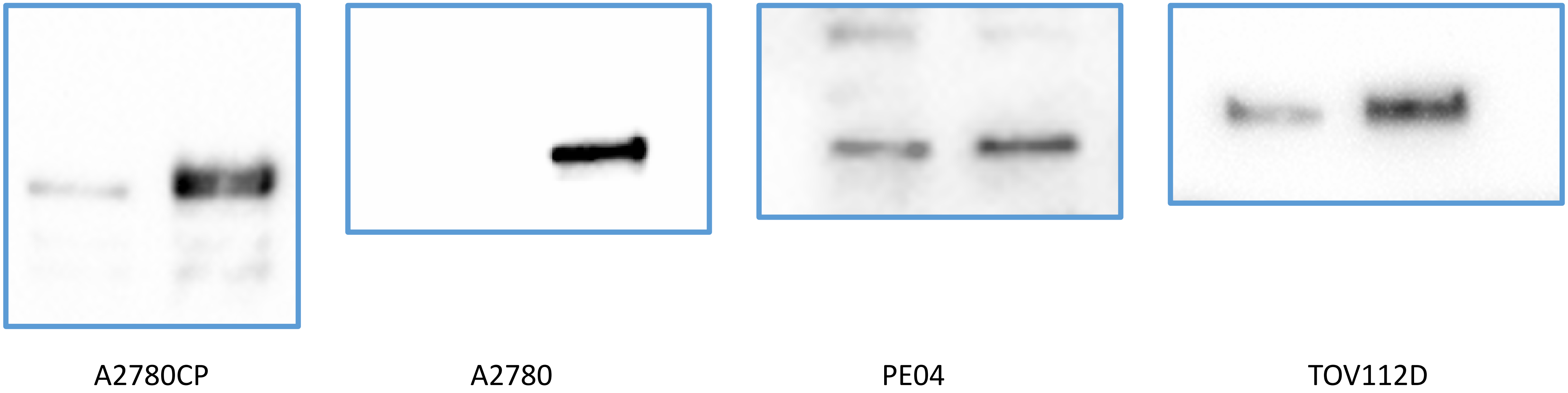

Supplementary Figure S10. Full-length blots representing the protein expressions of TIE-1, cleaved-PARP, and β-actin as shown in Figure 3c.

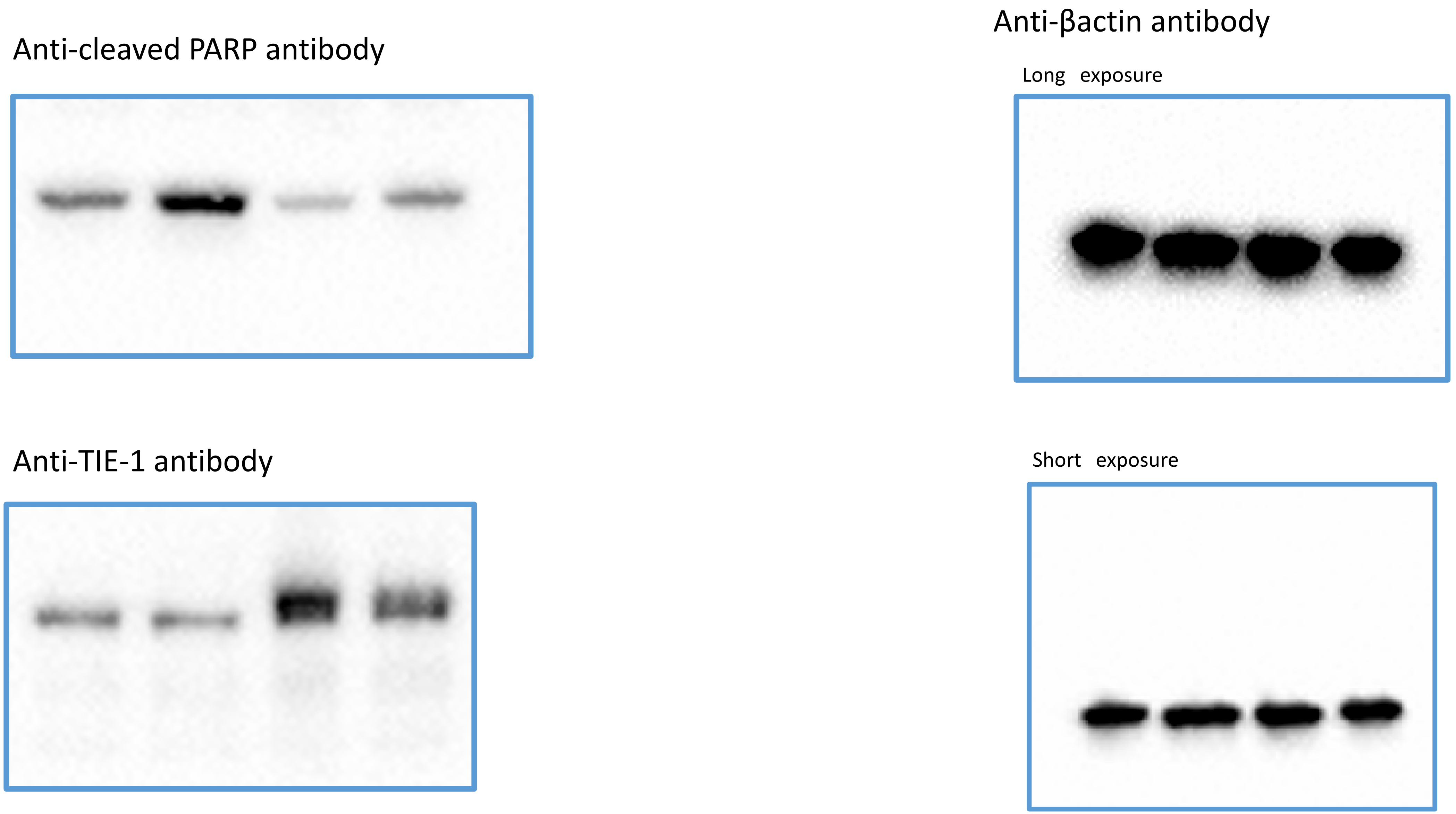

Supplementary Figure S11. Full-length blots representing the protein expressions of TIE-1, cleaved-PARP, and β-actin as shown in Figure 3d.

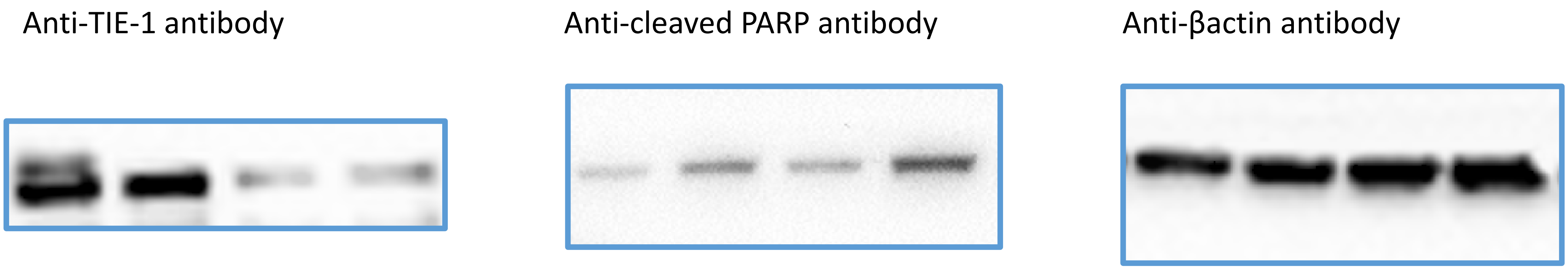

Supplementary Figure S12. Full-length blots representing the protein expressions of TIE-1, cleaved-PARP, and β-actin as shown in Figure 3e.

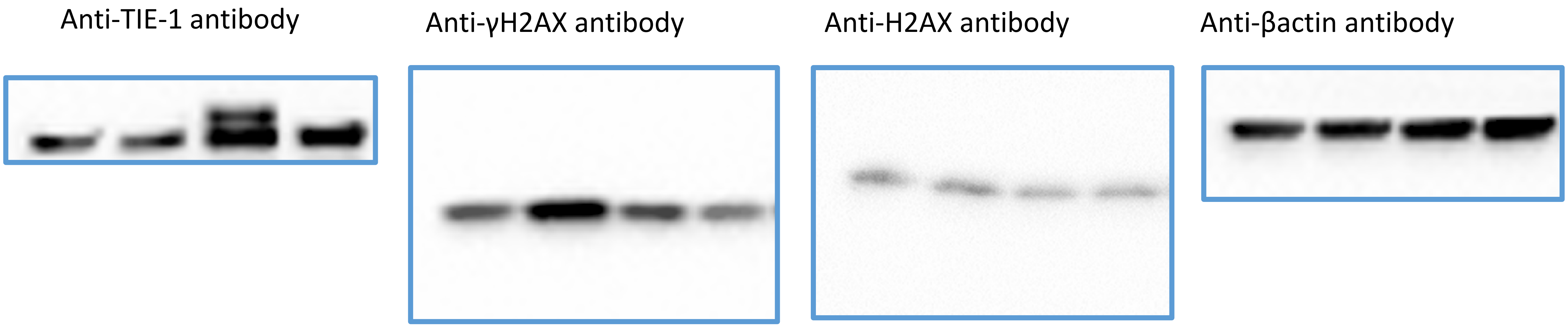

Supplementary Figure S13. Full-length blots representing the protein expressions of TIE-1, cleaved-PARP, and β-actin as shown in Figure 5b.

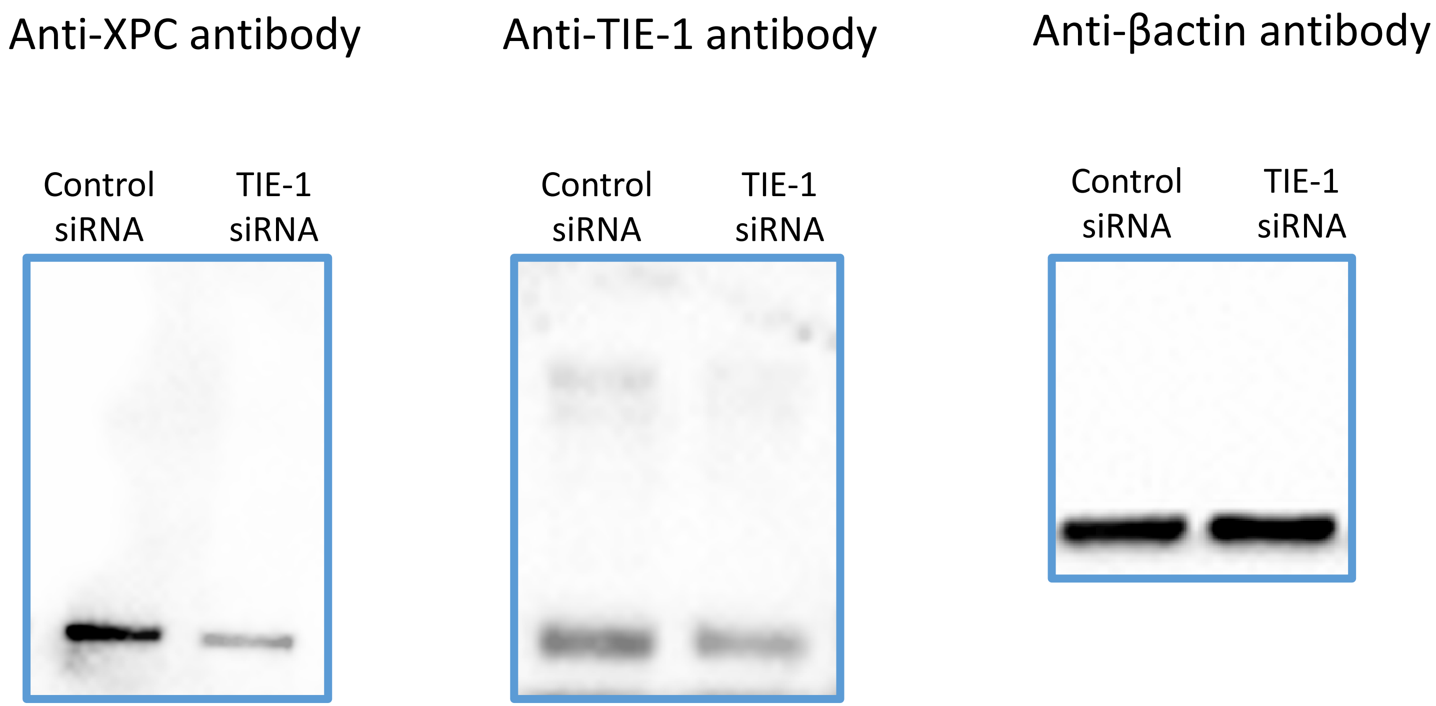

Supplementary Figure S14. Full-length blots representing the protein expressions of TIE-1, PARP, KLF5 and β-actin as shown in Figure 6a.

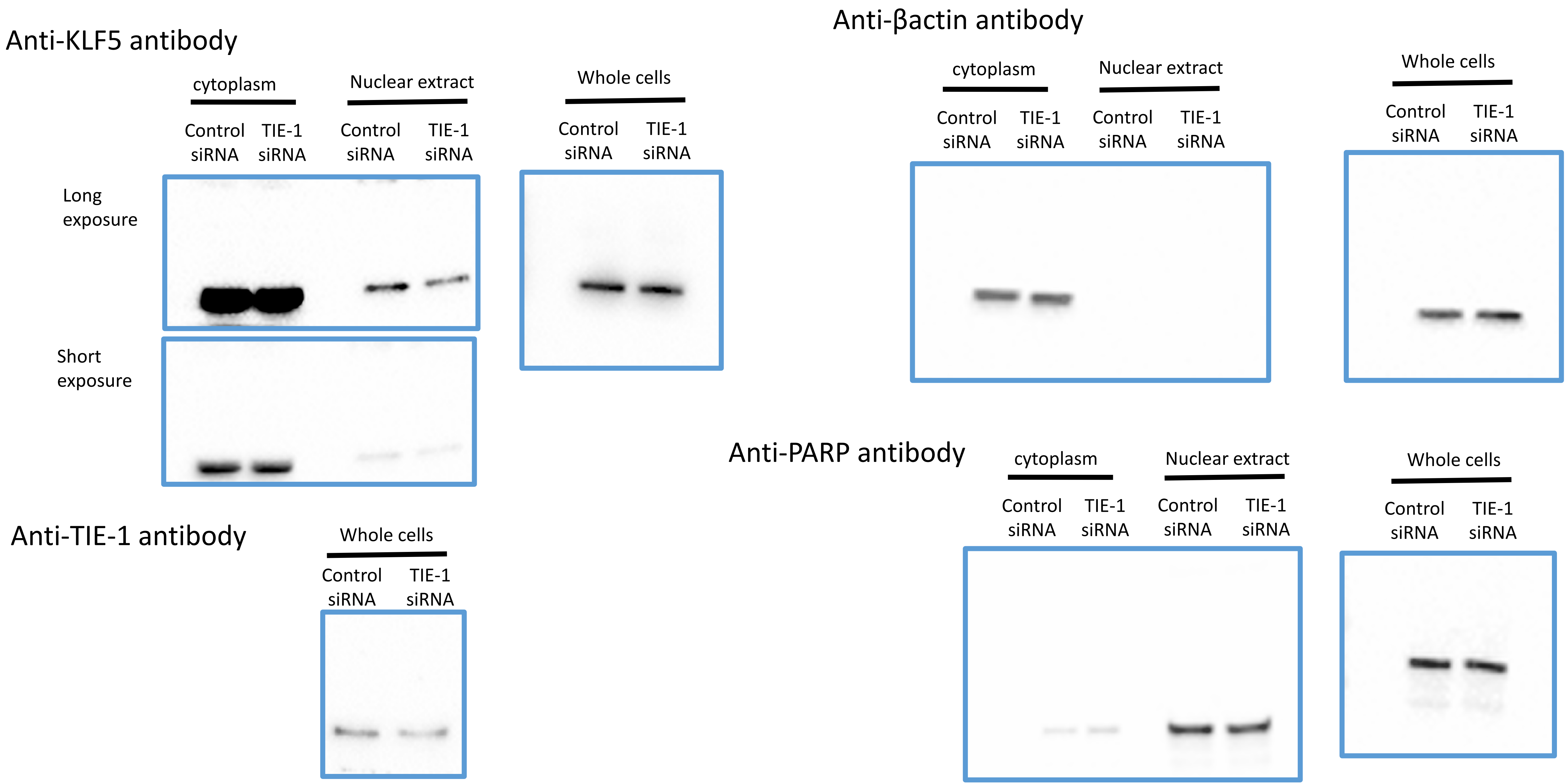

Supplementary Table 1: Patient characteristics

Demographic characteristics of the study population. *P* value, Pearson's chi-squared test for categorical variables and *t* test for continuous variables; SD, standard deviation; n, number of patients.

	**Platinum resistant , n (%)**	**Platinum sensitive, n (%)**	**Total, n**	***P* value**
Ovarian cancer stage				0.50
III	5 (56)	4 (44)	9	
IV	3 (75)	1 (25)	4	
Age, mean ±SD(years)	53 ±11	68 ±6	61 ± 10	<0.05

Supplementary Table 2: Hit selection groups and criteria.

Group[1]	Criteria[2]	Number of genes
FDR0.1	False Discovery Rate < 0.1	201
FDR0.2*	False Discovery Rate < 0.2 ∩<br>Differential viability > 20% ∩<br>Vehicle viability < 125% ∩<br>Fold change > 1.4	38
MAD	1 or more replicates scoring lower than 3 Median Absolute Deviations	101
PM5	Cisplatin viability < 85% ∩<br>Inclusion in top 5% of Predictive Model	30

[1] Genes may belong to multiple groups (e.g. FDR0.1 & MAD) - indicated as "Multi"
[2] Must satisfy all criteria

Supplementary Table 3: Heating programs of AAS

	Temperature (˚C)	Time (s)	gas	Flow (L/min)
Dry temperature	100	30	Ar	0.2
	300	15	Ar	0.2
Ash temperature	1200	20	Ar	0.2
Atomize temperature	2500	3	Ar	off
	2600	3	Ar	0.2
